# ADIPOCYTE-DERIVED FACTORS: IMPACT ON HEALTHSPAN AND LIFESPAN

**DOI:** 10.1093/geroni/igac059.1264

**Published:** 2022-12-20

**Authors:** Philipp Scherer

**Affiliations:** The University of Texas Southwestern Medical Center, Dallas, Dallas, Texas, United States

## Abstract

Adipocytes secrete numerous lipid and protein factors with profound effects on systemic energy homeostasis. One such adipokine that we first identified in the early 1990’s, adiponectin, has garnered significant attention as a potent mediator of insulin sensitivity and cell survival. FGF21 is another factor that is secreted by a number of cell types (including adipocytes), which has a beneficial effect on metabolism. Our group generated a novel mouse model that overexpresses, in an inducible fashion, physiological levels of FGF21 from adipocytes in the adult mouse. While comparable levels of constitutive overexpression of FGF21 from the liver do not have an impact on aging, adipocyte-derived FGF21 exerts a profound beneficial impact on health- and lifespan. This demonstrates that selective manipulation of adipose tissue per se has the potential to significantly reduce mortality and extend lifespan. The adipocyte-specific FGF21 transgenic animals have increased energy expenditure, weigh considerably less and exhibit an improvement in all systemic metabolic parameters examined to date. The mice further display unique immune-metabolic profiles of their adipose tissue depots, which defy the conventional changes associated with aging. Importantly, all these phenotypic alterations are achieved without a significant impact on adipose tissue beiging/browning. Moreover, at least some beneficial aspects of FGF21 appear to be mediated through a lowering of leptin, which leads to central leptin sensitization. Combined, these efforts shed additional light on the physiological effects of FGF21.

